# Stabilized quantum-enhanced SIEM architecture and speed-up through Hoeffding tree algorithms enable quantum cybersecurity analytics in botnet detection

**DOI:** 10.1038/s41598-024-51941-8

**Published:** 2024-01-19

**Authors:** Madjid G. Tehrani, Eldar Sultanow, William J. Buchanan, Malik Amir, Anja Jeschke, Mahkame Houmani, Raymond Chow, Mouad Lemoudden

**Affiliations:** 1 The George Washington University, Washington, DC USA; 2 Capgemini Deutschland GmbH, Berlin, Germany; 3 Blockpass ID Lab, Edinburgh Napier University, Edinburgh, UK; 4 Université de Montréal, Montreal, Canada

**Keywords:** Quantum simulation, Computer science, Software

## Abstract

For the first time, we enable the execution of hybrid quantum machine learning (HQML) methods on real quantum computers with 100 data samples and real-device-based simulations with 5000 data samples, thereby outperforming the current state of research of Suryotrisongko and Musashi from 2022 who were dealing with 1000 data samples and quantum simulators (pure software-based emulators) only. Additionally, we beat their reported accuracy of 76.8% by an average accuracy of 91.2%, all within a total execution time of 1687 s. We achieve this significant progress through two-step strategy: Firstly, we establish a stable quantum architecture that enables us to execute HQML algorithms on real quantum devices. Secondly, we introduce new hybrid quantum binary classifiers (HQBCs) based on Hoeffding decision tree algorithms. These algorithms speed up the process via batch-wise execution, reducing the number of shots required on real quantum devices compared to conventional loop-based optimizers. Their incremental nature serves the purpose of online large-scale data streaming for domain generation algorithm (DGA) botnet detection, and allows us to apply HQML to the field of cybersecurity analytics. We conduct our experiments using the Qiskit library with the Aer quantum simulator, and on three different real quantum devices from Azure Quantum: IonQ, Rigetti, and Quantinuum. This is the first time these tools are combined in this manner.

## Introduction

In the rapidly evolving digital landscape where cyber threats are growing both in sophistication and pervasiveness, maintaining robust cybersecurity measures has taken center stage. While traditional cybersecurity approaches remain effective to a degree, they often struggle to keep up with the constant flood of cyber attacks^1^. In recent years, machine learning has proven to be valuable in various cybersecurity applications. It’s been effective in tasks such as intrusion detection, malware classification, and anomaly detection by harnessing automated data analysis and pattern recognition capabilities^2^. Now, the rise of quantum computing is paving the way for even further improvements in cybersecurity analytics.

Quantum computing, renowned for its ability to perform intricate computations at a speed exponentially faster than traditional computers^3^, shows promising potential to revolutionize cybersecurity. Quantum machine learning, which has emerged as the intersection of quantum computing and machine learning, leverages the distinctive properties of quantum systems to devise innovative algorithms with the potential to outperform their classical counterparts^4^. In this paper, we explore the domain of quantum-enhanced cybersecurity analytics, with a special focus on employing quantum machine learning algorithms for botnet detection—a pressing cybersecurity issue with significant implications for network security^5^. By utilizing the power of quantum computing, we aim to establish a stable architecture and capitalize on the prospective speed enhancement offered by tree algorithms, thereby strengthening the effectiveness and efficiency of botnet detection methods.

The term Cybersecurity Analytics^6,7^ refers to the application of data analysis techniques to cybersecurity. Much of the literature on this subject takes a practical approach, offering tangible examples and implementable code for cybersecurity solutions^8–10^. However, a term that encapsulates cybersecurity analytics within the context of a quantum system is yet to be fully coined. This is a goal of our present work. In this paper, we introduce Quantum Cybersecurity Analytics (QCA) as a field that employs quantum technology, particularly quantum machine learning, to devise cybersecurity solutions.

We address the challenges and computational demands inherent to quantum machine learning algorithms through the creation of a stable architecture and the adaptation of the Hoeffding tree algorithm for incremental learning^11^. The current state of the art defined in Ref.^12^ shows the classification with a hybrid approach of 1000 data samples on a quantum simulator from a botnet dataset with an accuracy of 76.8%, whereas the total execution time is not reported. In their study, no signs of any real-device-based simulations or even computations on real quantum devices is shown. We outperform these achievements in the following ways:

1. We have extended the maximum sample size from 1000 to 5000 data samples in a quantum machine learning method, using real-device-based simulation through the quantum-enhanced Hoeffding Tree Classifier (QHTC) algorithm. Our method achieves an average accuracy of 91.2% and a final-round accuracy of 100%, all within a total computation time of 1687 s, which is on par with the total execution time observed in locally deployed quantum simulations.
2. Furthermore, and for the first time, we implemented various HQBCs on actual quantum devices. We managed to process a maximum of 100 randomly fixed data samples, achieving a top accuracy of 59.0%.

In addition, our work makes the following additional contributions:

1. We overcome the pitfalls due to the instabilities of long-running code on three different Azure Quantum Providers by code hardening.
2. We apply the batch-wise Hoeffding Tree algorithm instead of the usual loop-wise algorithms relying on gradient descent.
3. We compare a diverse set of binary classifiers on real devices, on real-device-based simulations as well as quantum simulators. All experiments are conducted consistently using the IEEE Botnet DGA dataset.
4. Quantum Cybersecurity Analytics is made possible.

The source code implementation is publicly available on GitHub^13^.

## Methods

This section emphasizes the experimental decisions made in this research. The first subsection covers the selection of quantum devices, real-device-based simulators, and quantum simulators utilized for conducting the experiments. The second subsection provides an explanation for the selection of the IEEE Botnet DGA Dataset, justifying its suitability for the analysis conducted in this research.

### Selected platforms

For this research, we opted to use a combination of real quantum devices, real-device-based simulators, and quantum simulators (pure software-based emulators) to reproduce the results reported in the study by Suryotrisongko et al.^14^, which focused exclusively on quantum simulators. Additionally, our experiments were conducted on three Azure Quantum Providers to expand the research scope beyond the utilization of IBM Quantum^14^. The real quantum devices we selected for our experiments were IonQ, Rigetti, and Quantinuum. To perform quantum simulations, we relied on the Qiskit SDK, utilizing Aer for simulations and real-device-based simulations.

The quantum computing configurations used in our experiments are presented in Table 1. The first column introduces a naming convention for referencing the platforms, facilitating better comprehension of the experimental results presented. Platforms functioning as real quantum devices are denoted by their respective names followed by the letter R. Platforms that combine real quantum devices with simulations, thereby serving as real-device-based simulators, are denoted by their names followed by the letter S.

**Table 1:** Naming conventions for selected platforms shown with their machine name and their device mode (quantum simulator, real-device-based simulator, or real quantum device).

Naming convention	Machine name	Device mode
Aer	Qiskit	Quantum simulator
IonQ-R	IonQ Aria	Real quantum device
IonQ-R	IonQ quantum simulator	Real-device-based simulator
Rigetti-R	Rigetti Aspen-M-3 with Qiskit	Real quantum device
Rigetti-S	Rigetti QVM	Real-device-based simulator
Quantinuum-R	Quantinuum H1-2^15^	Real quantum device
Quantinuum-S	Quantinuum H1-2 emulator^16^	Real-device-based simulator

### Description of the dataset

In this study, we evaluated our findings on DGA botnets using two datasets: the IEEE Botnet DGA Dataset^14,17^ and the UMUDGA dataset^18^. The UMUDGA dataset consists of 50 malware samples and is suitable for multiple classifications using HQBCs. However, for the purpose of comparing our results to^12^, we focused solely on the IEEE Botnet DGA Dataset in the current experiments. Nonetheless, the UMUDGA dataset may be considered for future investigations.

The IEEE Botnet DGA Dataset comprises a total of 1,803,333 data records. For our experiments, we randomly selected data samples from this dataset. Specifically, we used 1000 fixed random data samples for quantum simulators, following the approach in^12^, and real-device-based simulators. Additionally, we utilized 100 fixed random data samples for real quantum devices, and a separate set of 5000 fixed random data samples to test the new algorithm on real-device-based simulators.

As described in Ref.^12^, we extracted seven features from the analyzed domain names in the dataset. These features include:

1. *CharLength* The character length of the domain name.
2. *EntropyValue* The entropy value calculated using Shannon’s function with the probability distribution of characters in the domain name.
3. *RelativeEntropy* The distance or similarity of a domain name to the character probability distributions of either Alexa or DGA domain names, measured using the Kullback–Leibler divergence function.
4. *MinREBotnets* The minimum relative entropy with the domain names of DGA botnets.
5. *InformationRadius* The similarity or distance of a domain name to the domains of the ten botnet DGA families, calculated using the Jensen-Shannon divergence function.
6. *TreeNewFeature* A feature generated by a decision tree algorithm that combines the features Entropy, REAlexa, MinREBotnets, and CharLength to train a predictive model.
7. *Reputation* Provides information about the popularity and credibility of the website.

The summarized statistics for these features, including the mean, standard deviation, minimum, median, maximum, skewness, and kurtosis values, are presented in Table 2.

**Table 2:** Selected descriptive statistics of the IEEE Botnet DGA Dataset^14^ for the seven features according to the Anderson-Darling normality test.

Feature	Mean	StDev	Min.	Median	Max.	Skewness	Kurtosis
CharLength	17.20	6.82	4.00	16.00	73.00	0.81	0.02
EntropyValue	3.02	0.53	0.00	3.04	4.78	− 0.40	0.83
RelativeEntropy	1.66	0.82	0.20	1.55	10.10	1.63	6.91
MinREBotnets	1.28	0.57	0.00	1.23	5.99	0.84	1.24
InformationRadius	0.65	0.11	0.24	0.65	1.17	0.34	0.12
TreeNewFeature	0.45	0.34	0.00	0.35	0.99	0.38	− 1.52
Reputation	81.66	54.12	0.00	64.51	436.31	0.99	0.21

## Stable architecture for long-running experiments

This section discusses the issues encountered during long-running experiments and presents a stabilized architecture to address these problems. It includes the introduction of a new binary classifier and highlights relevant implementation issues.

### Reasons for instability

The current versions of Qiskit ML classifiers (qiskit-0.41.1 and qiskit-machine-learning-0.5.0), specifically Quantum Support Vector Classifier (QSVC), Primal Estimated sub-Gradient Solver for Support Vector Machines (Pegasos) QSVC, Variational Quantum Classifier (VQC), and Quantum Neural Network (QNN), have not been tested for compatibility with Azure Quantum Providers such as IonQ, Rigetti, and Quantinuum. Additionally, graceful exception handling has not been implemented. As a result, during the experimentation phase, we frequently experienced instability, including unexpected aborts and missing error messages in long-running notebook sessions. Code hardening revealed the following reasons for instability during experiments on real quantum devices:

**List 1: Reasons for instability**
1. Issues on the real quantum devices
  - Failure of a single circuit run causing a cascade effect regardless of progress.
  - Prioritization and scheduling bugs in the task queue.
  - Maintenance downtime.
  - Inability to deploy the quantum cloud architecture on a small scale due to insufficient or outdated documentation.
2. Issues with the hosted Jupyter notebooks in Azure Quantum workspace
  - Kernel failure.
  - Low memory.
  - Insufficient number of virtual CPUs.
  - Lack of visibility on progress and log processing.
3. Issues in the communication between real quantum devices and notebooks
  - Authentication and session failures.
4. Issues with the Jupyter Notebook on the client side
  - Termination after a maximum of 24 h, regardless of CPU or RAM power.
5. Issues related to different real devices
  - Deprecated application programming interfaces (API)s of Qiskit.
6. Issues stemming from the nature of the algorithm
  - Excessive number of loops.
  - Lack of code portability.
  - Inadequate exception handling.

We discovered that the stability of computing and network elements within the architecture is the primary limitation of cloud-based quantum computer delivery. However, none of our experiments on real quantum devices could last longer than three weeks. We were unable to establish a stable Transport Layer Security (TLS) connection and authentication for a 1000 random fixed data sample, leading us to select a reduced sample size of 100 random fixed data points for real quantum devices. The next subsection will present an architecture design that addresses points (1)–(5) in List 1 of instability reasons, followed by a subsection that will discuss necessary algorithmic changes to tackle point (6) in List 1. It is important to note that our experiments running on quantum simulators did not exhibit any instability.

### Stabilized architecture

**Figure 1:**
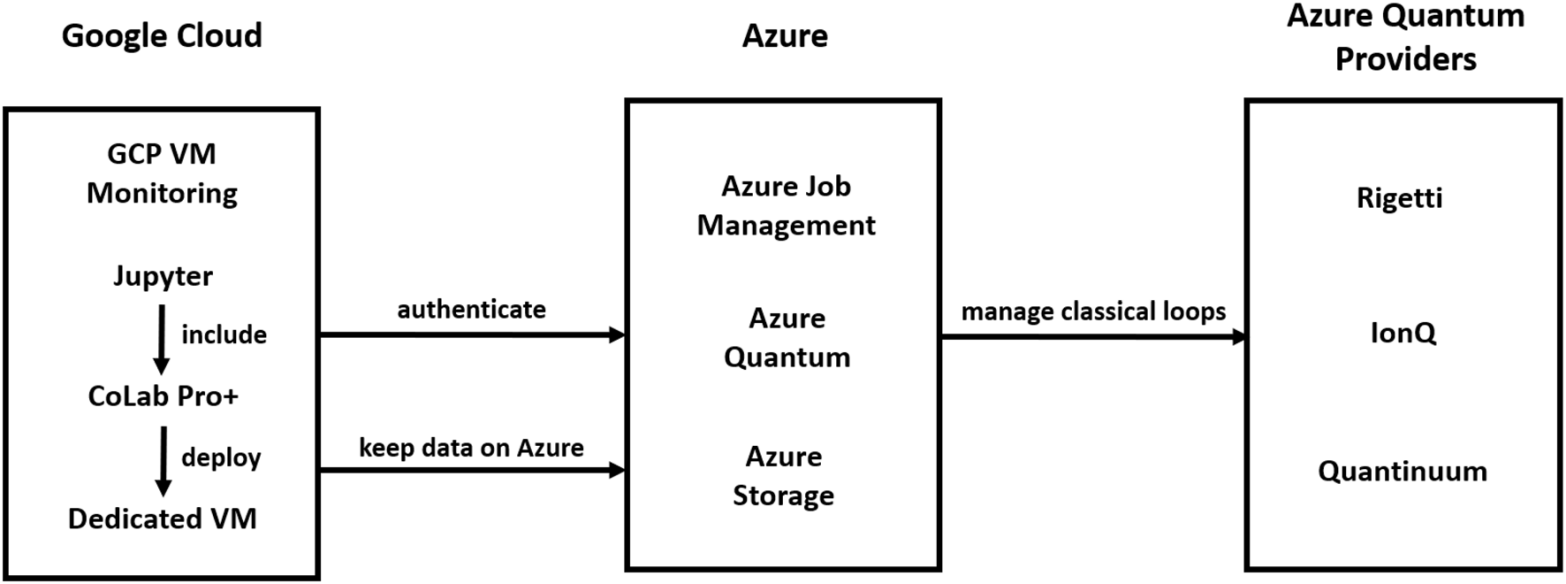
Stabilized architecture of experiments on real quantum devices comprising of three components Google Cloud, Azure and Azure Quantum Providers.

**Figure 2:**
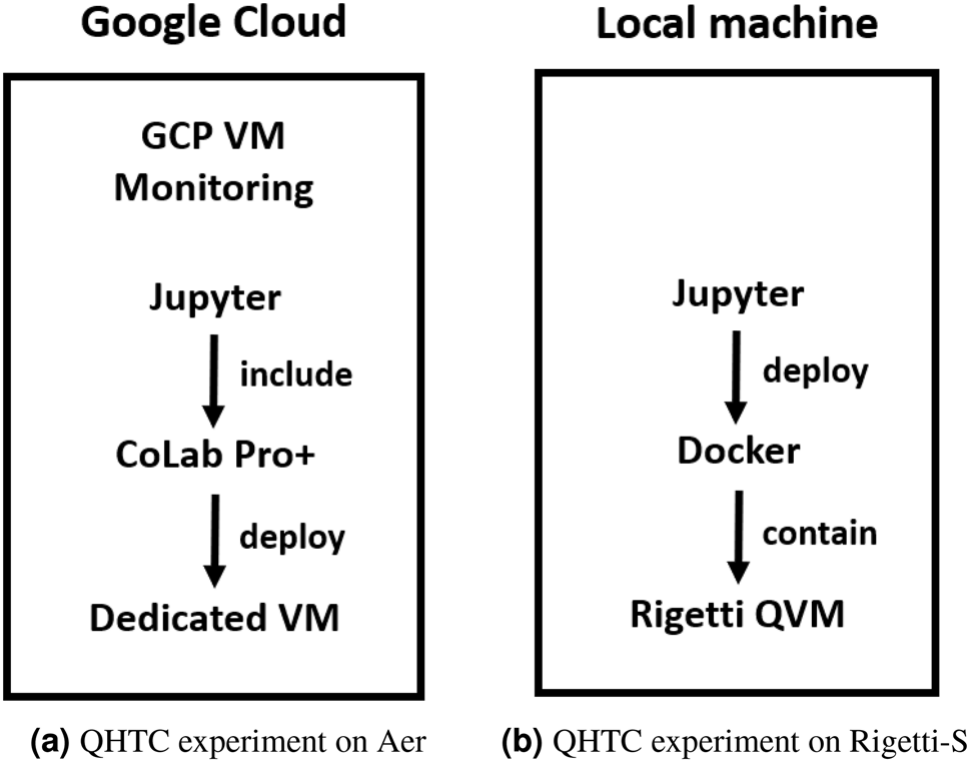
Stabilized architecture for QHTC experiments on quantum simulators Aer and Rigetti-S. The difference in implementation originates from differences in library functionalities available on Aer and Rigetti-S.

Our enhanced architecture design addresses the instability reasons (1)–(5) in List 1. The original architecture that led to instabilities consisted of an Azure real quantum device and an Azure component that involves an Azure Job Management, a storage account and an authentification component. The updated architectures introduce additional components to solve the instability issues mentioned in List 1. Experiments except QHTC are build on the architecture displayed in Fig. 1 and QHTC experiments apply the architecture in Fig. 2. Table 3 shows a series of steps that were executed to amend the instabilities.

**Table 3:** Steps taken to address the instabilities and the resulting influence on the instability number as displayed in List 1.

Action step	Instability that was reduced
Applying the save-load-continuous training (SLCT) technique frequently.	1a, 1c, 3a
Using tree-based algorithms instead of those requiring an optimizer.	6a
Selecting PyQuil (Rigetti) for container-based deployment to evaluate HQTC.	1d
Downsizing the sample size to 100.	1b, 6a
Using Colab Pro+ deployed on a dedicated Virtual Machine (VM).	2a, 2b, 2c, 4a
Implementing incremental learning.	6a
Utilizing logging and progress monitoring.	2d
Monitoring the session token and handling token refreshment.	3a
Continuous code review (paper code) and keeping API migration up-to-date frequently.	5a, 6b
Conducting code review (Qiskit code) and implementing exception handling.	1a, 1b, 6b, 6c

The architecture for experiments except QHTC includes a preceding step in a Google Cloud instance, where a Jupyter and Google Colab Notebook can be deployed on dedicated virtual machines to enable longer runtimes beyond the 24-h limit. The additional Jupyter Notebook facilitates the implementation of Qiskit code changes for exception handling specific to the algorithm and real quantum device. The Google Colab Pro+ Notebook provides stable runs for more than 1000 random fixed data samples. Additionally, a monitoring instance of a Google Cloud Platform (GCP) virtual machine with diverse logging capabilities aids in identifying, tracking, and resolving errors, including authentication and session failures.

HQML opens the door to a new generation of Security Information and Event Management (SIEM) systems known as quantum-enhanced SIEM (QSIEM). To illustrate the functioning of QSIEM, we present the first use case: defending against DGA botnet attacks for Distributed Denial of Service (DDoS) at the application layer using QSIEM. The integration of HQML with a robust SIEM like Azure Sentinel becomes highly beneficial at OSI-layer 7 (application layer), where HTTP and DNS traffic occur. This integration enables the detection of malicious domain names generated by DGA-Botnets for command-and-control servers, which are crucial for coordinating DDoS attacks. By identifying and blocking traffic associated with these domains, botnets can be prevented from receiving commands or initiating attack traffic.

**Figure 3:**
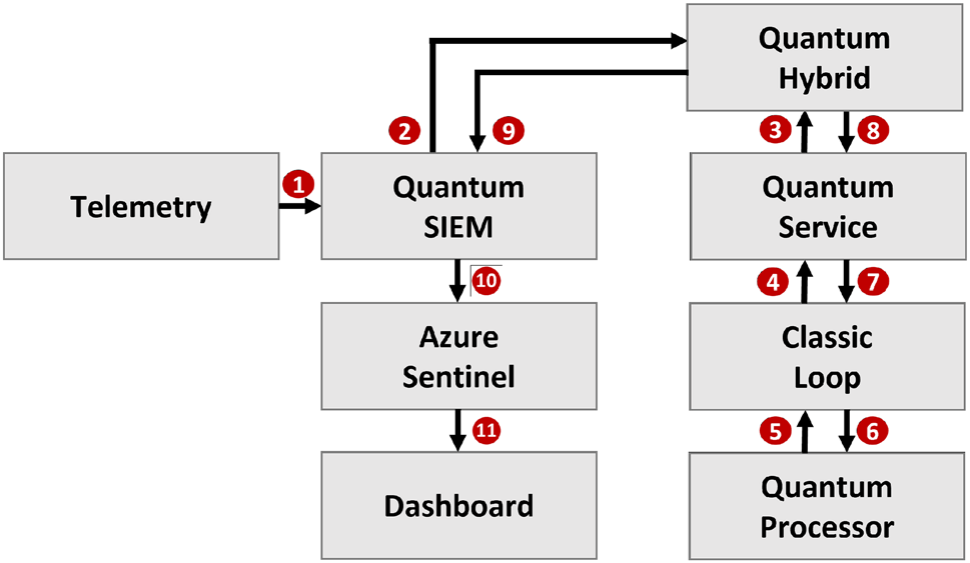
Quantum-enhanced SIEM: The individual steps are marked with numbers in red circles and are explained in List 2.

Our stabilized architecture aligns with the concept of a QSIEM solution. The steps in Fig. 3 are explained in List 2. Steps (2)–(9) are specific to training the HQML algorithm, while the productive algorithm utilizes telemetry input data to generate a classification using Quantum SIEM and Azure Sentinel, which is then displayed on the dashboard.

**List 2: Steps in the solution architecture**
1. Gather and preprocess the telemetry data required for the algorithm described in the next subsection.
2. Perform classic feature engineering as described in “Methods” section.
3. Deploy the algorithm for production use on Azure Quantum service.
4. Execute the entire circuit to and from the real quantum devices using the classical loop.
5. Collect all the results and accumulate the final output.
6. Save and update the classification algorithm.
7. Integrate the classification algorithm with Azure Sentinel.
8. Display the results of the classification algorithm to the user.

### Quantum-enhanced Hoeffding tree classifier (QHTC)

This subsection first describes the historical development of our scientific advances in the direction of the solution, followed by an explanation of the QHTC.

A realistic QCA solution, i.e., the QSIEM in the previous subsection, needs to be able to process online big data streaming. Hence, we sought an incremental approach to be applied to already known HQBCs. The most promising algorithmic candidate to reduce execution time and improve accuracy when executed on real-device-based simulators was the PegasosQSVC, in our opinion. Due to its stochastic gradient descent (SGD) optimizer, the PegasosQSVC performs fewer calculations by iterations and results in better generalization properties of the trained model than conventional gradient descent^19^. Instead of making the PegasosQSVC truly incremental, we applied a batch-wise strategy as an intermediate step between algorithms that need to process the entire training or test data samples at once and incremental algorithms.

**Figure 4:**
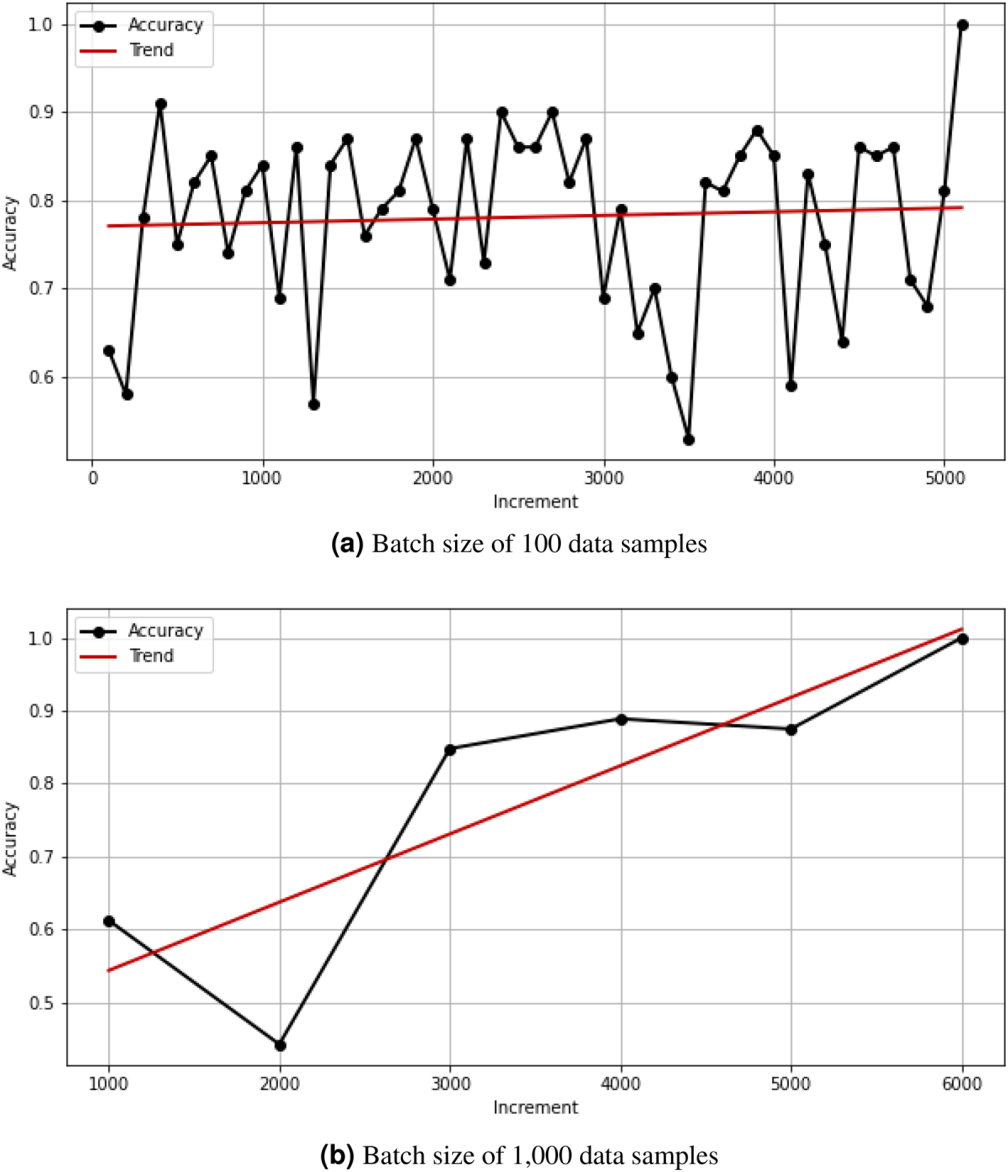
PegasosQSVC’s accuracy on quantum simulator AER with (a) a batch size of 100 data samples does not improve its accuracy with an increased number of batches, unlike with (b) a batch size of 1000 data samples.

The performance of PegasosQSVC with respect to accuracy development over time is displayed in Fig. 4 for batch sizes of 1000 as well as 100 random fixed data samples on the quantum simulator Aer. The PegasosQSVC shows good behavior in terms of accuracy increase with the number of batches if a batch size of 1000 data samples per batch is applied. But the real quantum devices are not able to handle 1000 data samples, but only 100 data samples per batch, as the results in Table 4 will show. In contrast, a batch size of 100 samples will not exhibit the appropriate increase in accuracy on real-device-based simulators or real quantum devices. Smaller batch sizes in the range of 100 data samples require a higher number (one magnitude) of circuits to be sent to the real quantum device, which will extend the execution time to an inappropriate level. This is the dilemma of limited data volumes in the Noisy Intermediate-Scale Quantum (NISQ) era.

Therefore, we decided to transition to a truly incremental algorithm and apply it batch-wise to reduce the number of shots sent to the real quantum device. The accuracy of a truly incremental algorithm will not suffer in this way. This was the breakthrough in terms of the algorithm’s accuracy and execution time on real-device-based simulators.

We found the algorithmic solution in a quantum-modified version of an incremental decision tree approach called the Hoeffding tree algorithm^20^. It is a generation algorithm for incremental decision trees that applies the Hoeffding bound^21,22^. The standard non-incremental version of the decision tree takes all data samples per leaf at once to compute a decision criterion per leaf. In contrast, the incremental version of a decision tree can process one data sample after another. The main advantage of this generation algorithm is that it guarantees, under realistic assumptions, the generation of an asymptotically arbitrarily similar incremental version of a decision tree compared to the same non-incremental version of the decision tree. Simultaneously, it maintains efficient computation speed. Additionally, the Hoeffding bound is independent of the probability distribution of the data samples. However, this implies the disadvantage that the Hoeffding bound, compared to distribution-dependent bounds, requires more data samples to reach the same level of similarity between the incremental version and non-incremental version of the decision tree.

We introduce the abbreviation HTC (Hoeffding tree classifier) for the original Hoeffding tree. Our quantum-modified version is called the quantum-enhanced Hoeffding Tree Classifier (QHTC), as presented in algorithm 3 and described below. QHTC is a batch-wise learning procedure that applies HTC with modified input data. We apply the HTC in an equivalent version following the HTC implementation of Ref.^23^ that is shown in Algorithms 1 and 2. The first step of QHTC is the mapping of the classical features of the input data to the quantum feature space using ZFeatureMap, although other mappings are also possible. Each feature column entry in the feature row represents a data point in quantum space (qubit) on the Bloch sphere and we want to measure the length of the cycle connecting all qubits per feature row. The reason is that the distance between two qubits represents a measure of how distinguishable they are. This cycle length is referred to as a ’quantum walk’ in the code.

The measurement of the cycle length relies on measuring the distance between two qubits on the Bloch sphere. For that, each qubit is converted via wave functions to its density matrix. These density matrices are listed in the same order as the classical feature columns and the trace distance of two density matrices is applied to measure the distance between two qubits that are neighbors on the cycle. The cycle length is determined by the order of data points in quantum space and, hence, by the order of the classical features given in the original data set. The determination of a distance metric that allows reordering of feature columns is left for future research. The initialization of HTC is performed accordingly.

The batch-wise computation of an incremental decision tree reduces the number of shots sent to the real quantum device drastically compared to usual loop-based optimizers, while not compromising its accuracy. This provides a solution to the instability reason (6a) mentioned in List 1. It allows us to deal with the realistic behavior of today’s real quantum devices that are prone to instability due to the noise problem inherent in today’s NISQ devices. The execution times and the accuracy benefit accordingly, as the results in the next section show in more detail.

**Algorithm 1:**
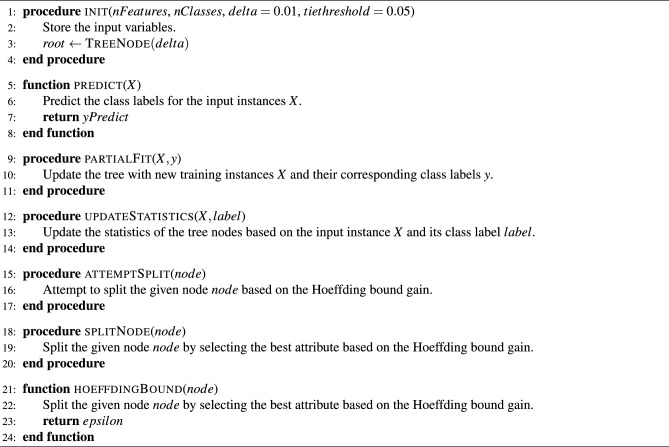
The HoeffdingTreeClassifier (HTC) following implementation^23^.

**Algorithm 2:**
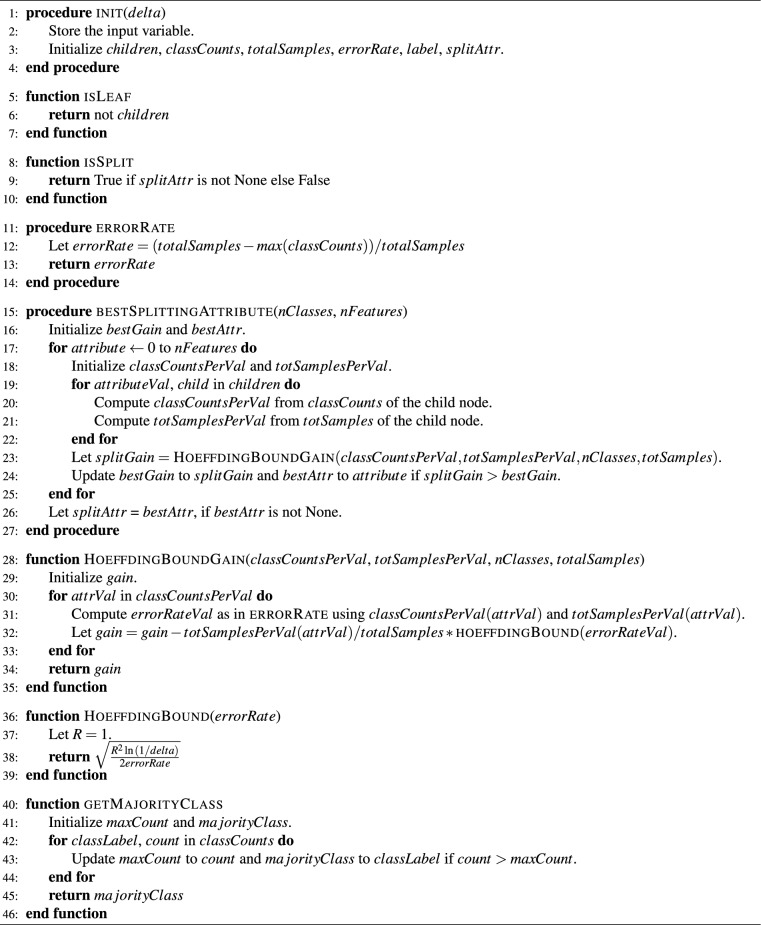
TreeNode (as part of Algorithm 1).

**Algorithm 3:**
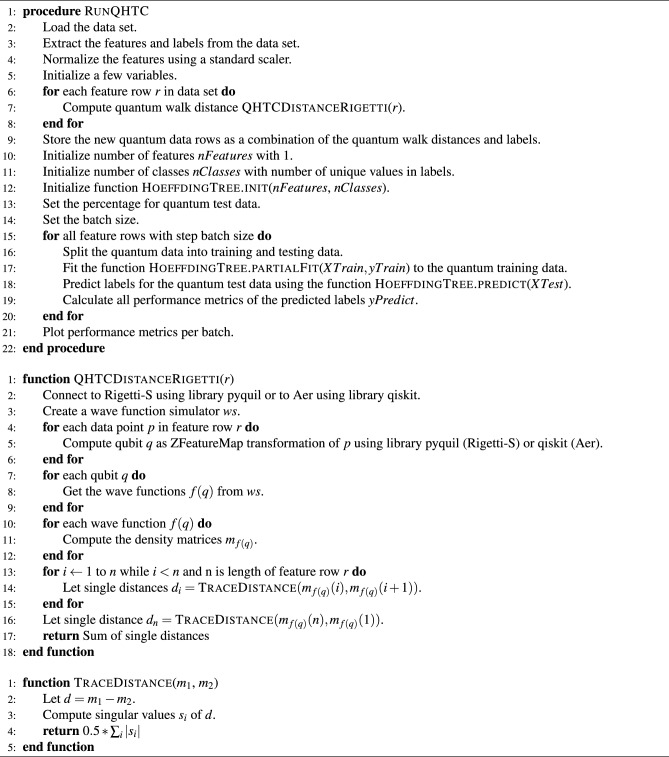
Batch-wise Learning with QHTC.

## Experimental results

The experimental results for different algorithms and quantum devices are presented in the following subsections, focusing on execution time, accuracy, and additional performance metrics for the QHTC algorithm.

### Execution time and accuracy

In this section, we present the experimental results for five different binary classifiers in terms of accuracy and execution time on quantum simulators, real-device-based simulators, and real quantum devices. The binary classifiers are VQC, PegasosQSVC, QSVC, Sampler Circuit of a QNN (SamplerQNN) and Estimator Circuit of a QNN (EstimatorQNN). Tables 4 and 5 showcase the accuracy, total computation time $\text {T}_{\text {total}}$, chosen feature map, and optimizer for various combinations of platforms and algorithms. The optimizer inherent in PegasosQSVC is the SDG, all other algorithms used the optimizer Constrained Optimization by Linear Approximation (COBYLA). The experiments on quantum simulators and real-device-based simulators were conducted with at least 1000 random fixed data samples, while the experiments on real quantum devices used 100 data samples due to computational limitations and instabilities. The source code implementation is publicly available on GitHub^13^ including a readme file showing the software library versions for all experiments.

**Table 4:** Performance results in terms of accuracy and total execution time $\text {T}_{\text {total}}$ of real quantum devices, using 100 data samples for all runs. For each algorithm and platform, the choice of the optimizer is also shown. The choice of feature map is ZFeatureMap for all experiments.

Algorithm and platform	Optimizer	Number of data samples	Accuracy (%)	$\text {T}_{\text {total}}$ (s)
VQC-IonQ-R	COBLYA	100	50	1,325,133
VQC-Rigetti-R	COBLYA	100	43	1,176,879
VQC-Quantinuum-R	COBLYA	100	44	972,732
PegasosQSVC-IonQ-R	SGD	100	41	156,156
PegasosQSVC–Quantinuum-R	SGD	100	44	972,732
PegasosQSVC-Rigetti-R	SGD	100	48	355,509
QSVC-IonQ-R	COBLYA	100	53	283,325
QSVC-Quantinuum-R	COBLYA	100	45	472,847
QSVC-Rigetti-R	COBLYA	100	39	385,153
SamplerQNN-IonQ-R	COBLYA	100	56	956,540
SamplerQNN-Quantinuum-R	COBLYA	100	46	1,087,789
SamplerQNN-Rigetti-R	COBLYA	100	53	1,601,895
EstimatorQNN-IonQ-R	COBLYA	100	59	1,165,819
EstimatorQNN-Quantinuum-R	COBLYA	100	50	1,167,143
EstimatorQNN-Rigetti-R	COBLYA	100	51	1,437,085

**Table 5:** Performance results in terms of accuracy and total execution time $\text {T}_{\text {total}}$ of quantum simulator and real-device-based simulator experiments, using 5000 data samples for QHTC, and 1000 data samples for all other algorithms. For each algorithm and platform, the choice of the optimizer is also shown. The choice of feature map is ZFeatureMap for all experiments. The QHTC achieves the accuracy result already after three out of five batches.

Algorithm and platform	Optimizer	Number of data samples	Accuracy (%)	$\text {T}_{\text {total}}$ (s)
QHTC-Rigetti-S	n.a.	5000	100	1687
VQC-Aer by^12^	COBLYA	1000	76.8	Not reported
VQC-Aer by^12^	RawFeatureVector	1000	84.4	Not reported
VQC-Aer	COBLYA	1000	54	4240
VQC-IonQ-S	COBYLA	1000	51	957,755
VQC-Quantinuum-S	COBYLA	1000	45	806,626
VQC-Rigetti-S	COBYLA	1000	46	889,708
PegasosQSVC-Aer	SGD	1000	90	45
PegasosQSVC-IonQ-S	SGD	1000	49	113,950
PegasosQSVC-Quantinuum-S	SGD	1000	49	174,416
PegasosQSVC-Rigetti-S	SGD	1000	55	206,729
QSVC-Aer	COBYLA	1000	87	3091
QSVC-IonQ-S	COBYLA	1000	50	178,529
QSVC-Quantinuum-S	COBYLA	1000	49	197,871
QSVC-Rigetti-S	COBYLA	1000	45	205,877
SamplerQNN-Aer	COBYLA	1000	76	374
SamplerQNN-IonQ-S	COBYLA	1000	59	746,992
SamplerQNN-Quantinuum-S	COBYLA	1000	48	852,774
SamplerQNN-Rigetti-S	COBYLA	1000	58	656,629
EstimatorQNN-Aer	COBYLA	1000	84	410
EstimatorQNN-IonQ-S	COBYLA	1000	63	780,480
EstimatorQNN-Quantinuum-S	COBYLA	1000	53	716,581
EstimatorQNN-Rigetti-S	COBYLA	1000	54	955,654

On real quantum devices, it is the first time that HQML algorithms run stable with 100 data samples. The PegasosQSVC performs well in terms of execution time due to its SGD optimizer which tends to converge a little faster than non stochastic optimizers. The PegasosQSVC stands out as the superior binary classifier. However, the algorithms in Table 4 don’t offer any quantum advantage over NISQ algorithms, whether in terms of time or cost improvement. The data samples size of 100 is an achievement on real devices, but this is not enough for the solution of real-life machine learning tasks of course. As the APIs of feature maps of Qiskit (see for example https://qiskit.org/documentation/stubs/qiskit.circuit.library.ZFeatureMap.html) have no endpoint to change the quantum real device, specific implementations are needed for each algorithm. Hence, we didn’t intend to compare QHTC over different quantum real devices. We left the implementation of additional coding routines in order to enforce specific real quantum devices and real-device-based simulators for future investigations.

On real-device-based simulators of quantum devices, it is the first time that a HQML algorithm run stable with 5000 data samples. All experiments reported by Ref.^12^ are conducted with the VQC algorithm and on the platform Aer. Among them, we show the one with the optimizer COBLYA, because we applied the same here, and with an optimizer (RawFeatureVector) that resulted in the maximal accuracy. Both experiments applied the variational form RealAmplitudes as we did in this study, where applicable. The PegasosQSVC on Aer exceeds the experimental results of Ref.^12^ in accuracy (90%) and in addition, it yields very good execution time (45 s). However, the QHTC algorithm outperforms all other binary classifiers in terms of accuracy, achieving perfect accuracy of 100% already after three out of five batches. The accuracy is discussed in more detail in the next subsection. Furthermore, QHTC exhibits significantly reduced total execution time (1687 s) of two orders of magnitude compared to other algorithms on real-devise-based simulators. QHTC make the cost of the second batch of 1000 data samples manageable. In contrast, other algorithms struggle to achieve the second batch. These algorithms would still require much longer calculation time if all conditions remain stable.

The experiments conducted on real-device-based simulators and real quantum devices are considered as a first step, and further improvements and specific implementations for each algorithm on different devices can be explored in future research. Overall, these results demonstrate that it is possible to construct superior algorithms for cloud-based NISQ deployments on real-device-based simulator Rigetti, achieving comparable execution times to quantum simulators while exceeding in terms of accuracy.

### Performance metrics of QHTC

We show the results of our QHTC (see algorithm 3) which is configured to run with five batches containing 1000 random fixed data samples each. We apply the feature map ZFeatureMap provided by Qiskit. Table 6 demonstrates achievements in terms of accuracy improvement. The increase in accuracy with the number of batches meets our expectations. We obtained an average accuracy of 91.2% and a final-round accuracy of 100% for QHTC already after three out of five batches. We used the same features and the same dataset as Ref.^12^ to be able to compare our results with theirs. These features are the same features that are available in the entire dataset itself. This may be the reason for such high accuracy. In future research, we can further improve the metric computation to avoid over-fitting and to make it more realistic by applying a PCA analysis as well as using a k-fold cross-validation per batch, with $k=10$ for example. In addition, the features EntropyValue and RelativeEntropy possess strong predictor properties for the entire dataset. Hence, the same issue will probably not happen to other datasets that don’t possess very strong predictor features.

**Table 6:** Metric results in terms of accuracy, F1-score and AUC for algorithm QHTC, displayed for five batches with 1000 data samples each and their average.

Batch	Accuracy (%)	F1-score (%)	AUC (%)
1	57.1	4.5	51.1
2	99.0	98.8	98.9
3	100.0	100.0	100.0
4	100.0	100.0	100.0
5	100.0	100.0	100.0
Average	91.2	80.7	90.0

## Conclusion and future work

Cybersecurity Analytics involves the collection of data to gather evidence, construct timelines, and analyze threats, thereby enabling the design and execution of a proactive cybersecurity strategy that detects, analyzes, and mitigates cyber threats. The next-generation Quantum Cybersecurity Analytics utilizes HQML to monitor network activity, promptly identify resource use or network traffic changes, and address threats. This advancement paves the way for a new generation of SIEM systems called quantum-enhanced SIEM (QSIEM). To illustrate how QSIEM operates, we presented the first use case of defending against DGA botnet attacks for DDoS at the application layer using quantum-enhanced SIEM.

As cybersecurity is built upon the analysis of amounts of big data, today’s NISQ era poses an obstacle for QSIEM for cybersecurity due to its inherent instabilities that enlarge with repeated and prolonged computations. This study found a way to overcome parts of the problem by proposing a new form of HQBCs that lead to significant improvements in the result’s accuracy as well as the algorithm’s execution times with real-device-based simulations compared to previous algorithms. The breakthrough was the application of a quantum-enhanced version of the incremental Hoeffding tree algorithm in a batch-wise version in order to take account of large amounts of incoming online stream data in addition to responding to the need for a reduced number of shots to the real quantum device. In addition to the improved accuracy, the experimental run times in real-device-based simulations were reduced drastically by three orders of magnitude to be in the same order as with the previous algorithms on the quantum simulator Aer that is deployed locally.

In general, the world of quantum simulators is much more beautiful than the world of computations on real quantum devices. This study showed for the first time that HQML algorithms were able to run stably with 100 random fixed data samples for several weeks on Azure Quantum Providers Rigetti, Quantinuum, and IonQ together with the library Qiskit. It is the first time these tools were combined. We achieved this by code hardening throughout the entire data flow process from the Jupyter Notebook to the real quantum devices, including all communications and algorithm-specific implementations of APIs per real quantum device. However, future research needs to build upon our progress in order to make the quantum computations on real devices stable for a much larger portion than 100 random fixed data samples, being just a very small fraction of the entire IEEE Botnet DGA Dataset. The enlargement of stability may also be pursued in the case of quantum simulations, as we only used a random fixed sample size of 1000 in the usual HQBC case and a random fixed sample size in the QHTC case when conducting real-device-based simulations.

Moreover, we left the implementation of additional coding routines in order to enforce all specific real quantum devices or real-device-based simulators in the case of the quantum-enhanced version as well as the original version of the Hoeffding tree algorithm for future investigations. In addition, the determination of a distance metric for QHTC that allows reordering of feature columns is left for future research. Our focus of this study in this regard was to show the excellent properties of these HQBCs for the DGA botnet classification problem in which we succeeded.

For future research, we also suggest investing more into PegasosQSVC because if we combine quantum supervised learning with rewarding and quantum reinforcement learning, we may have groundbreaking cybersecurity tools. Because current NISQ and hybrid models can support up to 5600 qubits, perhaps we don’t have a 5600 network feature in cyber data. Resulting from that, even in this NISQ period, we can probably make strong cyber use cases for existing quantum computers and HQML.

Furthermore, it is an open question as to what practical problem of which scientific fields the same approach of quantum-enhanced Hoeffding tree algorithms might apply as well. The UMUDGA dataset may be a next suitable choice for the DGA botnet detection field. We elaborated on a number of features of the IEEE Botnet DGA Dataset in order to give researchers from other fields a good starting point for their investigations.

## Data Availability

The datasets analyzed in this study are available in the Botnet DGA Dataset repository: 10.21227/rg6z-z622.
